# Construction and analysis for differentially expressed long non-coding RNAs and mRNAs in acute myocardial infarction

**DOI:** 10.1038/s41598-020-63840-9

**Published:** 2020-04-24

**Authors:** Ning Song, Xiang-Mei Li, Jun-Yi Luo, Hui Zhai, Qian Zhao, Xin-Rong Zhou, Fen Liu, Xue-He Zhang, Xiao-Ming Gao, Xiao-Mei Li, Yi-Ning Yang

**Affiliations:** 1 State Key Laboratory of Pathogenesis, Prevention and Treatment of High Incidence Diseases in Central Asia, Department of Cardiology, First Affiliated Hospital of Xinjiang Medical University, Urumqi, China; 2 Xinjiang Key Laboratory of Cardiovascular Disease Research, Clinical Medicine Institute, The First Affiliated Hospital of Xinjiang Medical University, Urumqi, China; 3 Xinjiang Key Laboratory of Medical Animal Model Research, Clinical Medical Research Institute of Xinjiang Medical University, Urumqi, China

**Keywords:** Long non-coding RNAs, Myocardial infarction

## Abstract

Long noncoding RNAs (lncRNAs) are transcripts longer than 200 nucleotides. Some lncRNAs are related to acute myocardial infarction (AMI) and can serve as blood-based biomarkers for AMI detection. To identify whether new lncRNAs participate in AMI, the expression of lncRNAs and mRNAs was analysed by microarray analysis (Agilent human array) with the limma package in R in two series: five paired peripheral blood mononuclear cell (PBMC) samples and four paired plasma samples from different AMI patients. In PBMCs, a total of 2677 upregulated and 458 downregulated lncRNAs were significantly differentially expressed; additionally, 1168 mRNAs were upregulated and 1334 mRNAs were downregulated between the AMI patients and controls. In plasma, we found 41 upregulated and 51 downregulated lncRNAs that were differentially expressed, as well as 9 mRNAs that were upregulated and 9 mRNAs that were downregulated among the two groups. Gene Ontology (GO) and Kyoto Encyclopedia of Genes and Genomes (KEGG) analyses were performed using the clusterProfiler package in R, and differentially expressed mRNAs were functionally annotated. The top differentially expressed mRNAs were associated with circadian rhythm, the NF-kB pathway, the p53 pathway and the metabolism pathway. We further performed target gene prediction and coexpression analysis and revealed the interrelationships among the significantly differentially expressed lncRNAs and mRNAs. The expression of four lncRNAs (uc002ddj.1, NR_047662, ENST00000581794.1 and ENST00000509938.1) was validated in the newly diagnosed AMI and control groups by quantitative real-time PCR (qRT-PCR). Our study demonstrated that the clustered expression of lncRNAs between PBMCs and plasma showed tremendous differences. The newly screened lncRNAs may play indispensable roles in the development of AMI, although their biological functions need to be further validated.

## Introduction

Acute myocardial infarction (AMI) continues to be the primary cause of disability and death, inducing a major health burden worldwide^1^. In the last decade, therapeutic methods, such as coronary intervention, coronary artery bypass surgery and medications, have ameliorated the prognosis of AMI, although its mortality remains nearly as high as before. Thus, it is critical to initiate the early identification and risk stratification of AMI, which would greatly accelerate early intervention^2,3^. Advances in genome-wide analyses, especially microarray profiling, play a potential role in discovering novel clinical biomarkers for AMI^4,5^. Recently, in the field of AMI, long noncoding RNAs (lncRNAs) have attracted extensive attention.

LncRNAs, ranging from 200 nucleotides to >10000 nucleotides in length^6^, lack protein coding capability since they have no open reading frames (ORFs). LncRNAs have been demonstrated to participate in specific physiological and pathological processes in a wide range of cardiovascular diseases (CVDs), such as heart failure^7–9^, cardiac hypertrophy^10^ and cardiometabolic diseases^11^, and are mainly related to epigenetic, transcriptional or posttranscriptional regulation.

Some studies have shown that lncRNAs could stably exist in peripheral blood mononuclear cells (PBMCs); for example, a lncRNA named OTTHUMT00000387022^12^ can serve as a unique biomarker to identify coronary artery disease (CAD), and ZFAS1 and CDR1AS^13^ also predict AMI with a higher specificity. With the goal of evaluating the ability of lncRNAs to serve as more intuitive and convenient biomarkers of cardiac development and pathophysiological processes, an increasing number of studies have shown that lncRNAs can be stable not only in the plasma but also in other body fluids^14^. Among some recent studies, several lncRNAs have been detected stably in the body fluids of patients, such as lncRNA SNHG1 and RMRP in plasma and lnc-PCDH9–13:1 in saliva, which can be biomarkers for lung cancer patients^15^ and early hepatocellular carcinoma (HCC)^16^, respectively. More excitedly, the expression levels of lncRNAs from plasma, such as HOTAIR^17^ and UCA1^18^, were altered in AMI. Therefore, we speculated that specific circulating lncRNAs can serve as AMI biomarkers at two levels, one in PBMCs and the other in plasma.

This study explored lncRNA and mRNA expression by microarray analysis, focusing on the roles of lncRNAs in AMI. In particular, we screened lncRNAs of clinical significance and validated their expression in different cohorts with or without AMI by qRT-PCR.

## Materials and methods

### Ethics and statements

This study was approved by the Research Ethics Committee of the First Affiliated Hospital of Xinjiang Medical University (Urumqi, China). All participants were enrolled in this study between December 2014 and September 2017 and provided written informed consent. In addition, all methods were performed in accordance with the relevant guidelines and regulations.

### Patients and sample collection

The diagnostic criteria of AMI were made in accordance with the 2016 American College of Cardiology (ACC)/American Heart Association (AHA) Guideline. All AMI patients were recruited from the Department of Cardiology, the First Affiliated Hospital of Xinjiang Medical University, from December 2014 to September 2017, and chest pain began within 12 h before undergoing percutaneous coronary intervention (PCI). Sex- and age-matched controls were consecutively recruited from the Health Physical Examination Centre of our hospital, and they had no evidence of cardiovascular or other systemic disease based on the records of physical examination, ECG and blood test. For all the subjects, the exclusion criteria were as follows: (i) thrombolysis therapy and other heart diseases (such as congenital heart disease, cardiomyopathy or severe valvular abnormalities); (ii) active inflammation; and (iii) renal or hepatic dysfunction, autoimmune diseases and tumours. A cohort of 59 AMI patients and 59 controls was finally enrolled in this study. To profile lncRNAs and mRNAs for microarray analysis in PBMCs, 5 AMI cases and 5 matched controls were randomly chosen and coded as PBMC-A1, -A2, -A3, -A4, and -A5 and PBMC-N1, -N2, -N3, -N4, and -N5. The other 4 AMI cases and 4 matched controls for chip analysis of plasma were randomly selected and coded as Plasma-A6, -A7, -A8, and -A9 and Plasma-N6, -N7, -N8, and -N9. The validation of dysregulated lncRNAs for microarray analysis was then analysed in the cohort of AMI patients (n  = 50) and controls (n  = 50). Blood samples (5 ml) collected from the peripheral veins of the patients were placed into test tubes containing EDTA before the administration of any anticoagulants.

### Collection and purification of mononuclear cells

Blood samples (including PBMCs and plasma) were collected within 2 hours after harvest, centrifuged at 3500 rpm for 10 min, and then composed of two layers. The upper plasma layer was directly used for plasma RNA extraction, and the lower layer was used for the lysis reaction. After mixing equal volumes of the lower blood cell layer and saline, we added Lymphocyte Separation Medium (TBD, Tianjin Biotechnology, Tianjin, China), which accounted for twice the total blood volume, to start the lysis reaction. Then, the layer was collected into a 1.5 ml EP tube and centrifuged at 10000 rpm for 5 min; thereafter, a white membrane was used as the white cell layer. After discarding the supernatant, the pellet remained at the bottom of the tubes.

### Plasma biochemical measurements

Total cholesterol (TC), triglycerides (TG), high-density lipoprotein cholesterol (HDL-C) and low-density lipoprotein cholesterol (LDL-C) were measured using a fully automatic biochemical analyser (AU5821, Beckman Coulter, USA).

### RNA extraction, labelling and array hybridization

TRIzol reagent (Invitrogen) was used to extract the total RNA from PBMCs. RNA was purified by the mirVana miRNA Isolation Kit (Ambion, Austin, TX, USA)^14^ based on the manufacturer’s instructions. The RNA quality and quantity from PBMCs were determined with a NanoDrop ND-1000 spectrophotometer (Thermo Fisher Scientific, Waltham, MA, USA). Only if the ratio of the absorbance at 260 nm and 280 nm (A260/A280) was between 1.8 and 2.1 could samples be used for follow-up steps. Plasma RNA was extracted and purified using the Qiagen serum/Plasma Kit (Qiagen #217184) following the manufacturer’s instructions; RNA was extracted from 400–500 µl of plasma and then put into 15 μl of diethylpyrocarbonate (DEPC) water to dissolve. No difference in the amount of extracted RNA in a unit of plasma was found between the control and AMI samples. Because of the particularity of plasma RNA, only concentrations >0.2 μg/μl could be applied to the reverse transcription reaction.

Sample labelling and array hybridization were implemented by the Agilent One-Color Microarray-Based Gene Expression Kit (Agilent Technology). In brief, the double-stranded cDNA was labelled with Cy5 and Cy3-dCTP (GE Healthcare) and then synthesized according to the protocol of CbcScript reverse transcriptase (CapitalBio). The transcription reactions of the dsDNA require T7 Enzyme Mix. The amplified cRNA was purified according to the manufacturer’s instructions of the RNA Clean-up Kit (MN). Before placing the mixture in an Agilent Hybridization Oven, a Klenow enzyme labelling strategy^19^ was used, and array hybridization was denatured.

### Validation of lncRNAs by qRT-PCR

Then, 11 μl of purified RNA from plasma or 1 μg of purified RNA from PBMCs was used for reverse transcription (Qiagen, China). According to the instructions of the Power SYBR Green PCR Master Mix (Applied Biosystems, USA), PCR with a 20 μl mixture (consisting of 10 µl of SYBR Green, 1 μl of primers, 7 μl of DEPC and 2.0 μl of cDNA) was performed in a 7900HT Fast Real-Time PCR system (Applied Biosystems, USA). The expression levels of lncRNAs were calculated via the comparative Ct (ΔΔCt) method and compared with those of the controls. GAPDH served as the internal control. The specificity of the PCR products was estimated by melt curve analysis. The primers for the lncRNAs in qRT-PCR are listed in Table 1.

**Table 1:** LncRNA primer design basic information table in AMI.

Gene name	Forward primer	Reverse primer	Hybridization temperature (°C)
uc002ddj.1	CTGCCCTGCTGACAGCTT	TCTCCGTGATGTTCTTGCGT	60
NR_047662.2	ACATCAGGCAAGACACAGGT	ACGGATAGGCACTTTGAGCTAC	60
ENST00000509938.1	CCCTGTTCTGACTTCAGGATCT	TGGGAAATGTGGCAATGTTGTT	60
ENST00000581794.1	CCCATTGGAGAGGACTGTGG	AGGGAATTTTCAGCCAAGGGTA	60

### Profiling of lncRNA expression

The total RNA of each sample was used to construct the lncRNA expression profiles. The expression of lncRNAs in PBMCs was represented using the Agilent human lncRNA + mRNA Array V4.0 (4*180 K format), which consisted of 41,000 lncRNA probes and 34,000 mRNA probes (Agilent, USA). Plasma lncRNA expression was analysed using the Agilent SBC Human ceRNA Array (4*180 K format) (GPL22120) (https://www.ncbi.nlm.nih.gov/geo/query/acc.cgi), which contained 68,423 human lncRNAs, 88,371 circRNAs and 18,853 human mRNAs (Agilent, USA).

### Mapping and identification of differentially expressed genes

CLUSTER 3.0 software with an adjusted data function was used to preprocess the data, for example, by performing log2 transformation, median centering and average linkage clustering. The lncRNA and mRNA array data were normalized and summarized by GeneSpring software V13.0 (Agilent, USA). Volcano plot filtering, scatter plot filtering and hierarchical clustering can screen out the different classifications of lncRNA and mRNA expression in the form of images. The implementation of tree visualization was performed with Java Treeview (Stanford University School of Medicine, Stanford, CA, USA).

### GO and KEGG pathway and disease analyses

Gene Ontology (GO) analysis (http://www.geneongoloty.org/) was performed to illustrate the unique biological significance of the differentially expressed genes^20^. The potential functions of the genes were divided into three categories: cellular components, molecular functions and biological processes. Based on the current Kyoto Encyclopedia of Genes and Genomes (KEGG) database, crucial pathways and related diseases were identified by KEGG pathway (http://www.genome.jp/kegg/) and disease (http://www.genome.jp/kegg/disease/) analysis. Fisher’s exact test and the chi-square test were applied to identify the significant GO terms and KEGG data, and the standard of threshold values was formulated by the false discovery rate (FDR) and *p*-value.

### Protein-protein interaction network construction

A protein-protein interaction (PPI) network could serve as a tool to assess the interactive relationships of the differentially expressed mRNAs, and one was constructed through the Search Tool for the Retrieval of Interacting Genes/Proteins (STRING, http://string.embl.de/)^21^ database with a combined score greater than 0.4 as the cut-off criterion. In addition, GO and KEGG enrichment analyses were performed by STRING. Visualization was carried out with the reliable online bioinformatics software Cytoscape v3.5.1 (Cytoscape Consortium, New York, NY). To acquire more meaningful mRNAs, the related analysis parameters used were as follows: species: Homo sapiens; active prediction methods: all; and degree centrality greater than one was set as a cut-off criterion.

### Construction of the regulatory co-expression network

The network of coding-noncoding coexpressed genes was constructed with the biological functions, especially to recognize the novel and significant genes. Correlations between lncRNAs and their corresponding mRNAs were calculated with Pearson’s correlation, and coefficients not less than 0.95 were used to draw the coexpression network through Cytoscape v 3.5.1 software. The degree centrality was used to determine the links, wherein a higher degree score indicates a more central location within the network.

### Target gene prediction

Since the potential functions of most lncRNAs have not been well annotated, they were predicted on account of the related *cis-* and *trans-*mRNAs. *Cis*-regulated target genes were selected according to the distance of 10 kb near the coding gene location, while LncTar^22^ was used to assume the *trans*-regulated target genes mainly in view of the different expression levels. Pearson correlation coefficient >0.99 and *p* < 0.05 were used for target gene prediction.

### Statistical analysis

The data were analysed with IBM SPSS Statistics 22.0 software (SPSS Inc., Chicago, Illinois, USA) and GraphPad Prism 6 (GraphPad Software). For continuous variables, the data are presented as the mean ± standard deviation (SD) and were compared using either the Mann-Whitney U test or the Kruskal-Wallis test as appropriate. The chi-square test or Fisher’s test was used for categorical variables. The relative expression levels of lncRNAs between the two groups were calculated via independent-sample t-tests. GO and KEGG pathway analyses were evaluated using Fisher’s exact test. For all statistical analyses, a two-tailed *p* < 0.05 was regarded as significant.

## Results

### Study cohorts

This study recruited 59 AMI patients and 59 matched healthy controls. The expression profiles of lncRNAs and mRNAs from 9 AMI patients and 9 matched controls were detected using microarrays, and the remaining participants were used for validation. The detailed clinical and demographic characteristics of the 18 study participants are summarized in Table 2.

**Table 2:** Clinical and phenotypical characteristics of 9 patients and 9 matched controls.

Characteristic	Matched Controls	AMI patients
Age, years	52.4 ± 3.22	53.8 ± 4.79
BMI, kg/m^2	23.8 ± 3.77	25.1 ± 2.96
Systolic blood pressure, mmHg	118.7 ± 11.39	120.2 ± 14.45
Diastolic blood pressure, mmHg	80 ± 10.75	80.3 ± 11.11
Heart rate, beats per minute	76 ± 7.14	81 ± 8.77
Creatinine, umol/L	82.1 ± 9.14	79.35 ± 15.14
Glucose, mmol/L	4.91 ± 1.14	7.76 ± 2.96*
TG, mmol/L	1.89 ± 0.69	2.73 ± 2.79*
TC, mmol/L	3.83 ± 0.71	4.59 ± 0.44*
LDL, mmol/L	3.48 ± 0.29	3.89 ± 0.54*
HDL, mmol/L	0.82 ± 0.12	1.25 ± 0.04*

The data are presented as the mean ± standard deviation. BMI: body mass index. TG, triglycerides; TC, total cholesterol; LDL, low density lipoprotein. HDL, high density lipoprotein; t-test, *p < 0.05 compared with matched controls.

### LncRNA and mRNA expression profiles in the PBMCs of AMI

**Figure 1:**
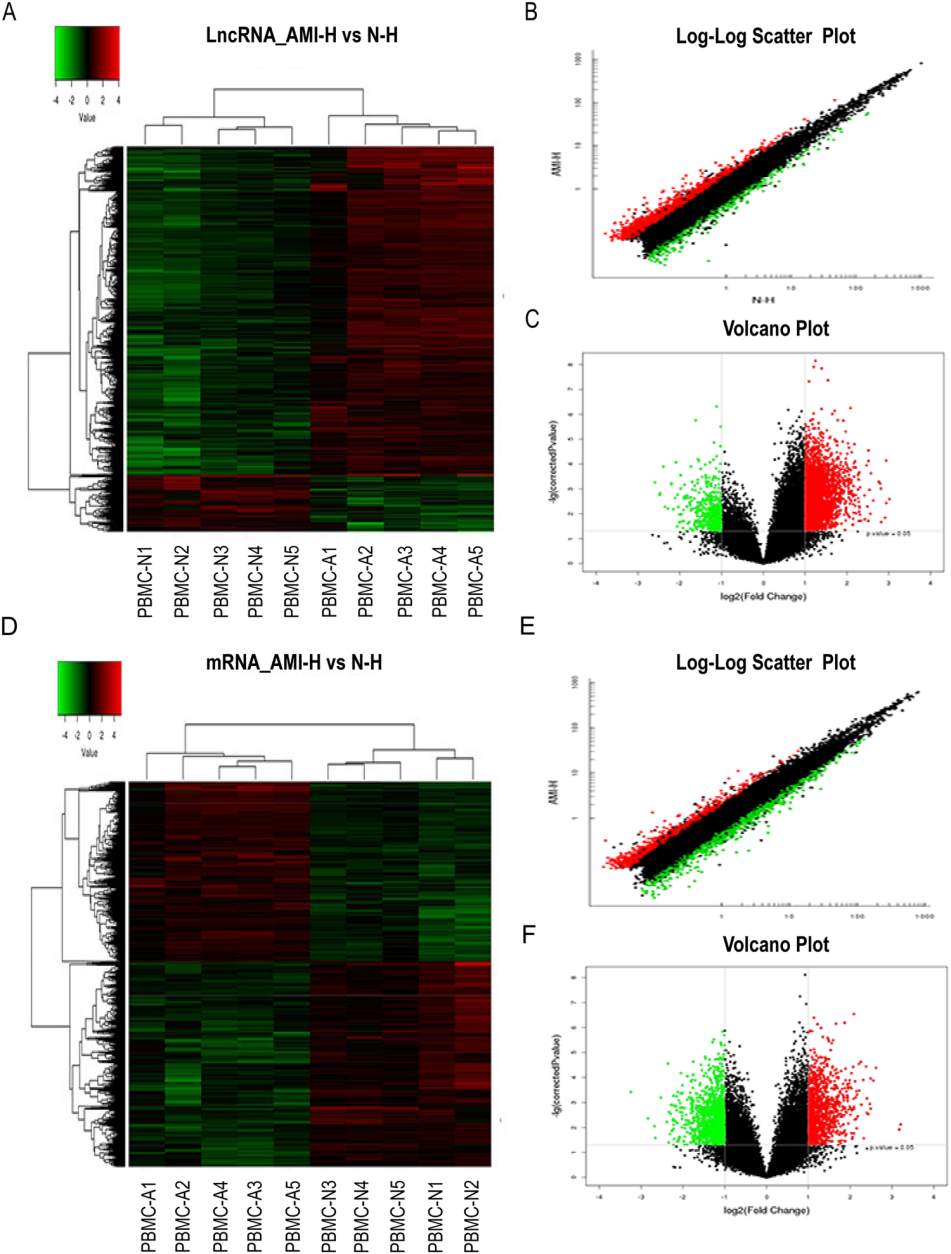
Differential expression of lncRNAs and mRNAs in AMI and matched controls in PBMCs. (**A**) Expression values are represented through hierarchical clustering analysis in red and green, indicating up-regulated and down-regulated lncRNAs expressions in AMI (PBMC-A1, PBMC-A2, PBMC-A3, PBMC-A4, PBMC-A5) and (PBMC-N1, PBMC-N2, PBMC-N3, PBMC-N4, PBMC-N5), respectively. (**B**) Scatter plot of differential lncRNAs expression. (**C**) Volcano plot of differential lncRNAs expression. **D**. Expression values are represented through hierarchical clustering analysis and indicated up-regulated and down-regulated mRNAs expressions in AMI (PBMC-A1, PBMC-A2, PBMC-A3, PBMC-A4, PBMC-A5) and (PBMC-N1, PBMC-N2, PBMC-N3, PBMC-N4, PBMC-N5), respectively. (**E**) Scatter plot of differential mRNAs expression. (**F**) Volcano plot of differential mRNAs expression. The red and green spots indicate up-regulation and down-regulation, respectively. X-axis: log2 Fc; Y-axis: −1×log10 (corrected p-value) for each probe.

Hierarchical clustering analysis showed that the expression levels of lncRNAs and mRNAs were significantly different in AMI patients compared with the matched controls. Of the 41,000 lncRNAs detected on the microarray, 3135 lncRNAs were found to be differentially expressed in AMI, including 2677 upregulated and 458 downregulated lncRNAs. In the scatter plot and volcano plot, every dot represents a lncRNA, the red dots represent the upregulated lncRNAs (fold change (Fc)>2, *p* < 0.05), the green dots represent the downregulated lncRNAs (Fc < –2, *p* < 0.05), and the black dots represent the remaining lncRNAs (–2<Fc<2, *p* > 0.05) **(**Fig. 1A–C). The top 20 differentially expressed lncRNAs according to the Fc values are presented in Supplementary Table 1. Among the 34,000 detected mRNA probes, 1168 were upregulated and 1334 were downregulated by the criteria of FDR-corrected *p* < 0.05 and absolute Fc>2. The top 20 distinctively expressed mRNAs according to the Fc values are presented in Supplementary Table 2. Every dot represents a lncRNA, the red dots represent the upregulated lncRNAs (Fc>2, *p* < 0.05), the green dots represent the downregulated lncRNAs (Fc < –2, *p* < 0.05), and the black dots represent the remaining lncRNAs (–2<Fc<2, *p* > 0.05) **(**Fig. 1D–F).

### Differentially expressed lncRNAs and mRNAs in the plasma of AMI

**Figure 2:**
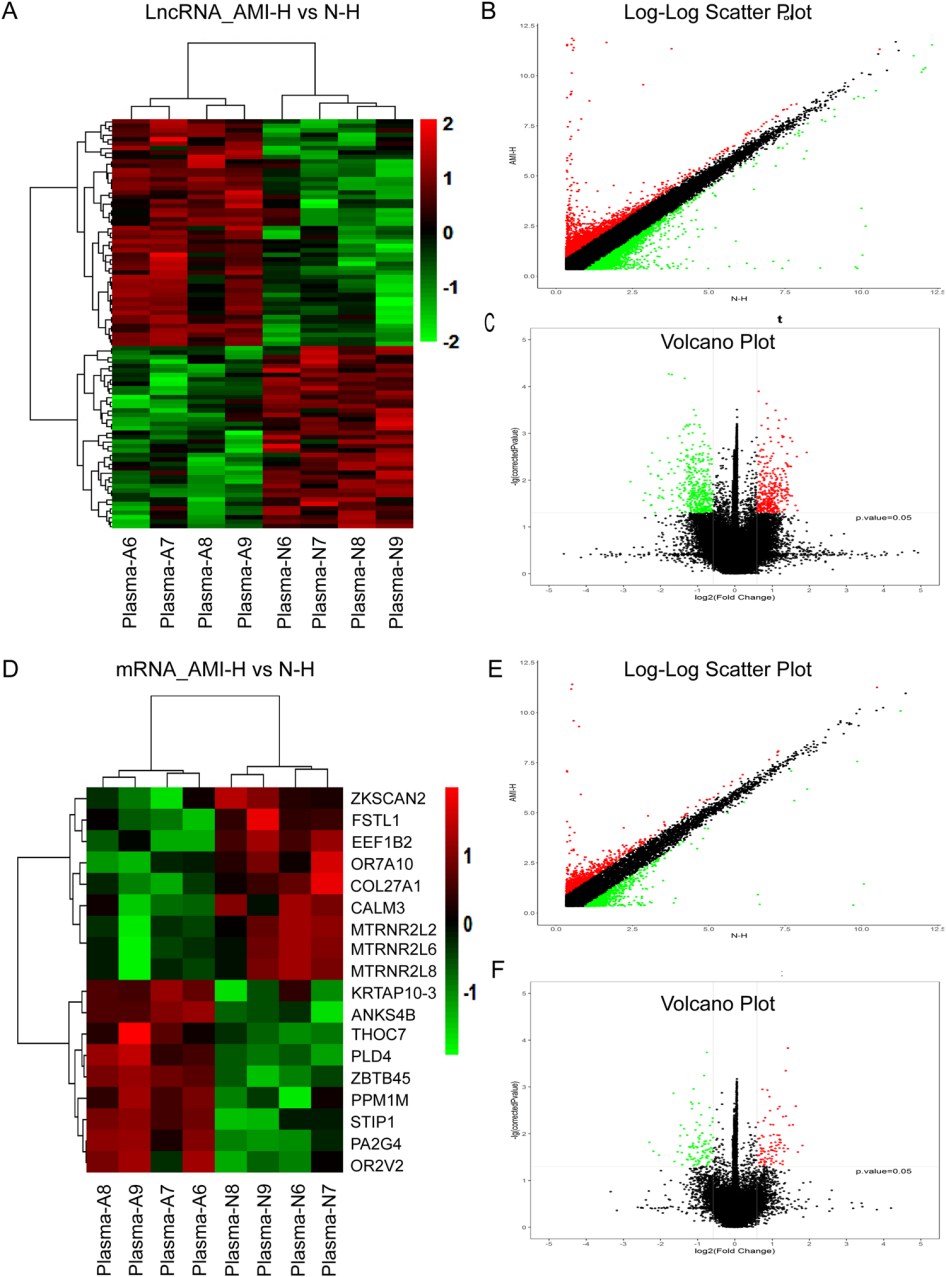
Differential expression of lncRNAs and mRNAs in AMI and matched controls in plasma. (**A**) Expression values are represented through hierarchical clustering analysis in red and green, indicating up-regulated and down-regulated lncRNAs expressions in AMI (Plasma-A6, Plasma-A7, Plasma-A8, Plasma-A9) and (Plasma-N6, Plasma-N7, Plasma-N8, Plasma-N9), respectively. (**B**) Scatter plot of differential lncRNAs expression. (**C**) Volcano plot of differential lncRNAs expression. (**D**) Expression values are reresented through hierarchical clustering analysis and indicated up-regulated and down-regulated mRNAs expressions in AMI (Plasma-A6, Plasma-A7, Plasma-A8, Plasma-A9) and (Plasma-N6, Plasma-N7, Plasma-N8, Plasma-N9), respectively. (**E**) Scatter plot of differential mRNAs expression. (**F**) Volcano plot of differential mRNAs expression. The red and green spots indicate up-regulation and down-regulation, respectively. X-axis: log2 Fc; Y-axis: −1 × log10 (corrected p-value) for each probe.

Additionally, the locus-by-locus lncRNA probes from the plasma of AMI patients (n = 4) and matched controls (n = 4) were examined by microarrays, and all results are presented following the criteria of FDR-corrected *p* < 0.05 and absolute Fc>1.5. We found 41 upregulated and 51 downregulated lncRNAs after hierarchical clustering and scatter and volcano plot analysis **(**Fig. 2A–C**)**. The top 20 differentially expressed lncRNAs according to the Fc values are presented in Supplementary Table 3. We also found that 9 mRNAs were upregulated and 9 mRNAs were downregulated in AMI patients **(**Fig. 2D–F**)**. The details of the 18 differentially expressed mRNAs are presented in Supplementary Table 4.

### Basic characteristics of lncRNAs in the PBMCs of AMI

**Figure 3:**
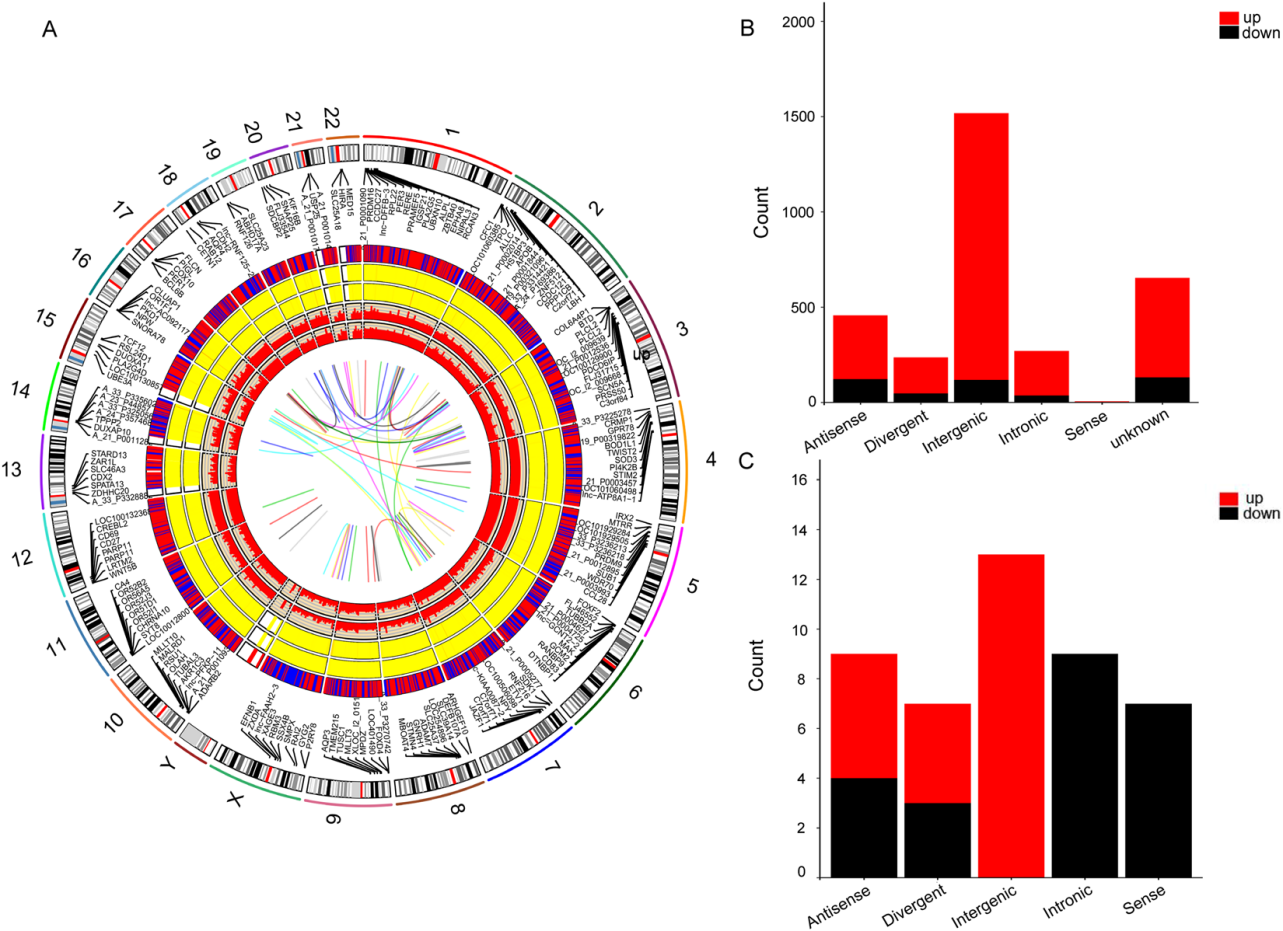
Identification of differentially expressed lncRNAs in AMI. (**A**) RCircos plot showing lncRNAs and mRNAs on human chromosomes in PBMCs. From the outside in, the first layer of the RCircos plot is a chromosome map of the human genome, black and white bars are chromosome cytobands, and red bars represent centromeres. In the second circle, the mRNAs which the degree >7 in the coexpression network are labeled. In the third layer, all differentially expressed lncRNAs and mRNAs are marked in red and blue. The fourth and fifth layers represent the mean expression values of significantly differentially changed lncRNAs and mRNAs in AMI and control groups. The sixth circle shows all differentially expressed lncRNAs and mRNAs according to Fc>2.0 and *p* < 0.05. The innermost circle indicates the degree of the labeled transcripts. The network in the center of the plot represents the core network; red lines indicate the linked transcripts in the same chromosome, blue in different chromosomes. (**B**) Venn diagram presents the lncRNAs counts and categorizations in PBMCs. (**C**) Venn diagram presents the lncRNAs counts and categorizations in plasma.

The basic characteristics of all differentially expressed lncRNAs and mRNAs in PBMCs, which are widely distributed in all chromosomes covering chromosomes X and Y, are shown in the Circos plot **(**Fig. 3A**)**. The Venn diagram indicates the lncRNA counts and types according to the differentially regulated lncRNA categorizations. Next, we classified the distinctive PBMC lncRNAs into five categories^23^ according to the genomic loci of their neighbouring genes **(**Fig. 3B**)**. Although 590 lncRNAs were not successfully categorized, the well-annotated lncRNAs were classified into the following five categories: intergenic (40.42%), antisense (14.61%), intronic (8.61%), bidirectional (7.53%), and sense (0%). Moreover, the plasma lncRNAs were the most abundant in the intergenic (44.57%), sense (19.57%) and intronic (18.48%) categories versus the antisense (9.78%) and bidirectional (7.61%) categories **(**Fig. 3C**)**.

### Functional analysis of differentially expressed genes in PBMCs in AMI

**Figure 4:**
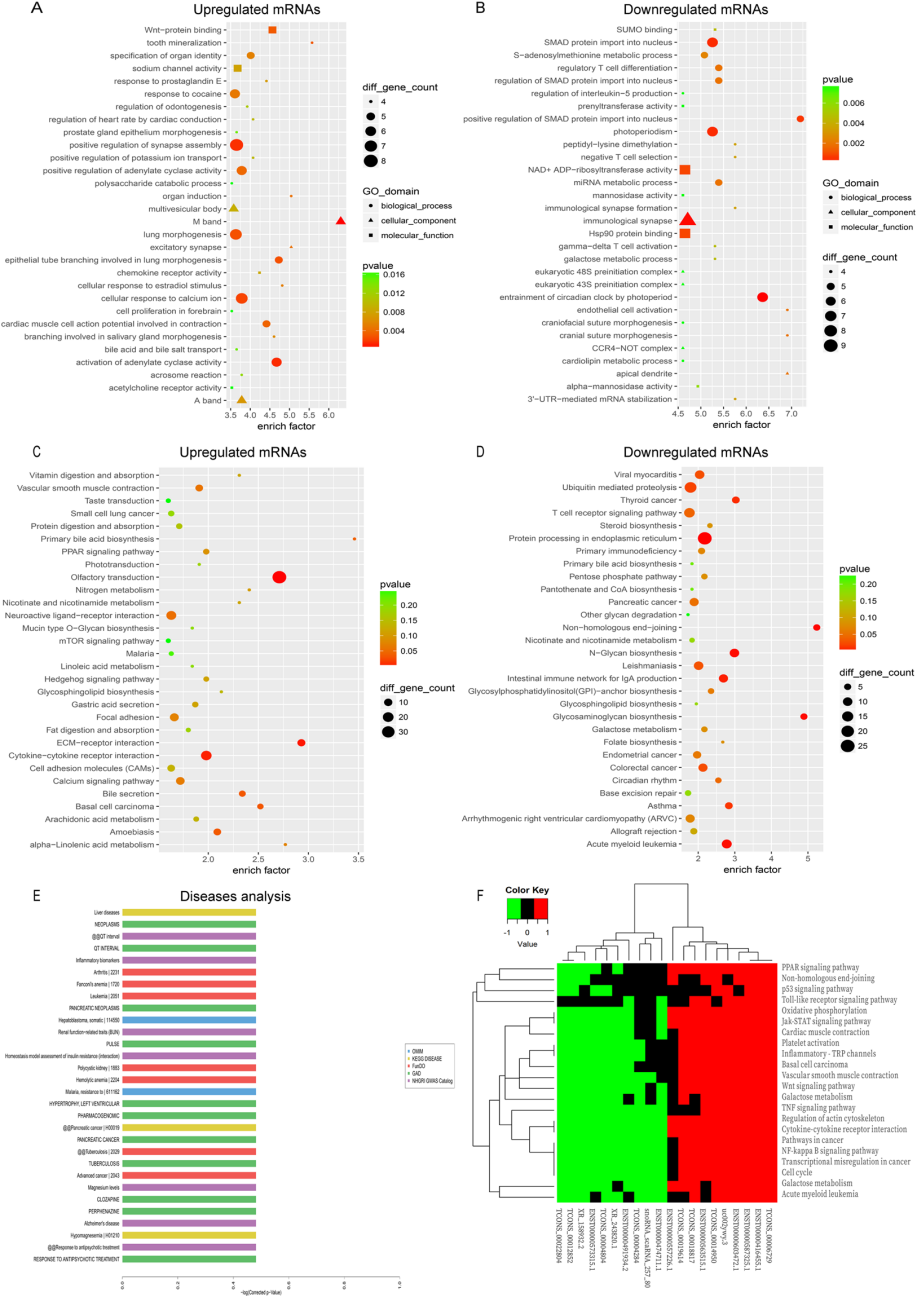
GO and KEGG analyses of mRNAs in AMI. (**A,B**) GO annotations of up- and down-regulated mRNAs with top 30 enrichment scores of biological processes. (**C,D**) KEGG enrichment pathway analysis of up- and down-regulated mRNAs with top 30 enrichment scores. (**E**) Disease analysis of up- and down-regulated mRNAs with top 30 enrichment scores. The disease terms derived from five database: OMIM, KEGG disease, FunDO, GAD, NHGRI GWAS Catalog, which were indicated in blue, yellow, red, green, purple, respectively. (**F**) KEGG annotation for the top 20 lncRNAs via co-expression of their mRNAs functions. Hierarchical clustering analysis of 20 lncRNAs that were differentially expressed in the KEGG pathway terms. X-axis on the left: 10 lower expressed lncRNAs in green. X-axis on the right: 10 higher expressed lncRNAs in red, X-axis on the middle: no significantly expressed lncRNAs in black.

After GO analysis, we found that the most enriched GO terms were involved in endothelial cell activation, cardiolipin metabolic process, regulation of heart rate by cardiac conduction, cellular response to calcium ion and cardiac muscle cell action potential involved in contraction **(**Fig. 4A,B**)**. KEGG pathway analysis showed that the differentially expressed mRNAs were mostly enriched in viral myocarditis, the mTOR signalling pathway^24^, circadian rhythm, arrhythmogenic right ventricular cardiomyopathy (ARVC), and the metabolism pathway^25^ (Fig. 4C,D). In the disease analysis, the distinct mRNAs were enriched in several diseases, and the top 30 diseases are shown in Fig. 4E.

To intuitively clarify the links of the expressed lncRNAs with functions and genes, we then visualized the hierarchical clustering results, as shown in Fig. 4F, which presents a clearer image to view the relationships between the top 10 upregulated and 10 downregulated lncRNAs and pathways through the enrichment analysis of the coexpressed mRNAs. A total of 20 lncRNAs were related to the occurrence and development process of heart diseases, such as cardiac muscle and vascular smooth muscle contraction and platelet activation. The most enriched pathways corresponding to the dysregulation of lncRNAs related to heart diseases were the peroxisome proliferator-activated receptor (PPAR) signalling pathway, NF-kappa B signalling pathway, p53 signalling pathway and Janus kinase signal transducer 2 and activator of transcription (JAK/STAT) signalling pathway.

### PPI network analysis of mRNAs in the PBMCs of AMI

**Figure 5:**
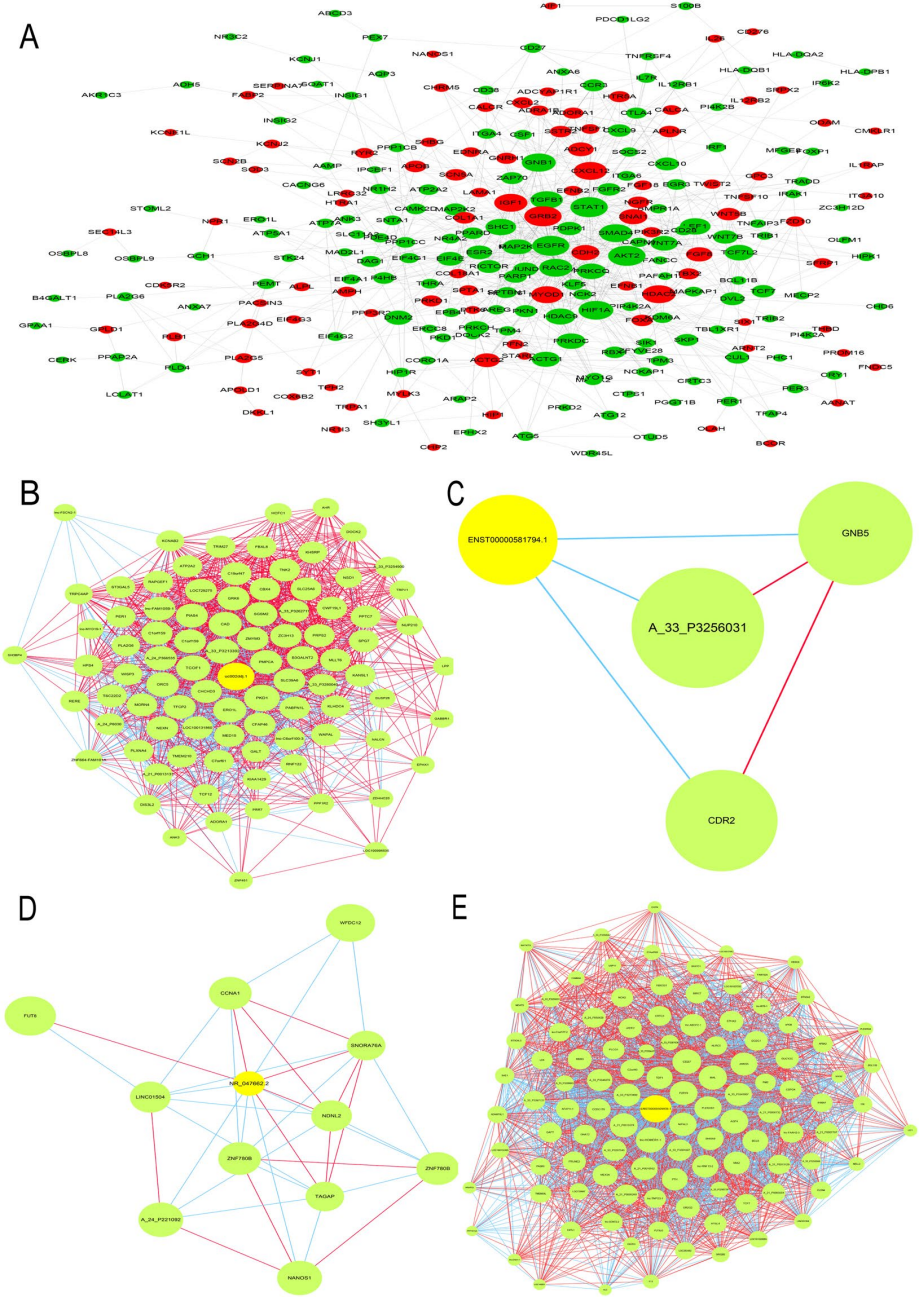
PPI and lncRNA-mRNA coexpression network. (**A**) PPI Network Analysis of significantly up- and down-regulated genes. Up-regulated genes were labeled in red, down-regulated genes were labeled in green. (**B**–**E**) LncRNA-mRNA network was constructed based on the correlation analysis between the lncRNAs (uc002ddj.1, ENST00000509938.1, ENST00000581794.1, and NR_047662.2) and mRNAs. In the network, yellow node represents the lncRNAs, and green node represents the target mRNAs. The red and blue outgoing links represent the up-regulation and down-regulation, respectively. The size of node represents the correlation strength.

According to the GO and KEGG enrichment analyses of mRNAs associated with cardiovascular development and progression, almost 200 mRNAs were screened out through the STRING database and a PPI network was constructed. Visualization was performed with Cytoscape v3.5.1. As shown in Fig. 5A, several PPI nodes had high connectivity degrees: EGFR (degree=37), TGFB1 (degree=24), RAC2 (degree=23), AKT2 (degree=25), HDAC2 (degree=24), CXCL12 (degree=22), and STAT1 (degree=22).

### lncRNA-mRNA coexpression network in the PBMCs of AMI

Coexpression networks, which contain lncRNAs and their corresponding mRNAs, facilitate the intricate network and can play potential roles in AMI progression. Therefore, we specifically selected 4 lncRNAs (ENST00000509938.1, ENST00000581794.1, NR_047662.2 and uc002ddj.1) mainly because of the potential functions of their coexpressed mRNAs. The details of these four lncRNAs are listed in Table 3. Their coexpressed mRNAs, including PKD1, LOC729275, TCOF1, KANSL1, TFCP2, GRK6, OR2D3, AFAP1L1, A_21_P0010378, lnc-HOMER1–1, AQP4, lnc-MYB-1, ADAMTSL1, and BIRC7, which are related to AMI occurrence and development, are shown in Fig. 5B–E. The details of these coexpression networks are listed in Table 4.

**Table 3:** The 4 lncRNAs related information in AMI.

Gene name	*p*	FC	Regulation	Probe ID	Chr	Start	End	class
uc002ddj.1	0.012	2.011	down	p33784	16	15226577	15229270	Intronic
NR_047662.2	0.032	2.014	down	p25499	8	12394587	12523120	Intergenic
ENST00000509938.1	0.021	2.818	up	p25499	8	12394587	12523120	Intergenic
ENST00000581794.1	0.026	2.067	up	p14886	7	30606019	30634385	Antisense

**Table 4:** LncRNA-mRNA network analysis.

Source	Target	Correlation	*p*	Target. GeneSymbol
p33784 (uc002ddj.1)	A_23_P200001	−0.960559547	1.01E-05	NEXN
	A_21_P0014030	0.966696721	5.17E-06	KANSL1
	A_24_P112750	0.962211388	8.52E-06	TFCP2
	A_33_P3224891	0.973992788	1.94E-06	LOC729275
	A_33_P3292915	0.967364222	4.77E-06	TCOF1
	A_21_P0011418	0.997661702	1.30E-10	PKD1
p25499 (NR_047662.2)	A_23_P68436	−0.96254	8.23E-06	WFDC12
	A_24_P354724	0.954042	1.85E-05	TAGAP
	A_33_P3629585	0.96346	7.46E-06	SNORA76A
p29378 (ENST00000509938.1)	A_32_P167239	0.969288887	3.75E-06	AFAP1L1
	A_33_P3420757	0.952945885	2.03E-05	AQP4
	A_33_P3264188	0.971385427	2.83E-06	OR2D3
	A_21_P0010378	0.960644564	1.00E-05	A_21_P0010378
	A_21_P0004563	0.956834201	1.44E-05	lnc-HOMER1–1
	A_21_P0004689	0.950426377	2.49E-05	lnc-MYB-1
	A_33_P3343007	−0.955769436	1.59E-05	A_33_P3343007
	A_23_P362183	−0.965730495	5.79E-06	ANKS6
	A_23_P8834	−0.952022439	2.19E-05	EPHX2
	A_23_P17134	−0.958736807	1.21E-05	MAL
	A_33_P3256031	−0.955699815	1.60E-05	A_33_P3256031
	A_33_P3308332	−0.96910919	3.84E-06	PLEKHB1
p14886 (ENST00000581794.1)	A_23_P205778	−0.908336667	0.000276167	GNB5
	A_33_P3256031	−0.904670509	0.000321602	A_33_P3256031
	A_24_P339071	−0.903667925	0.000334929	CDR2

### Validation of dysregulated lncRNAs in the PBMCs of AMI

**Figure 6:**
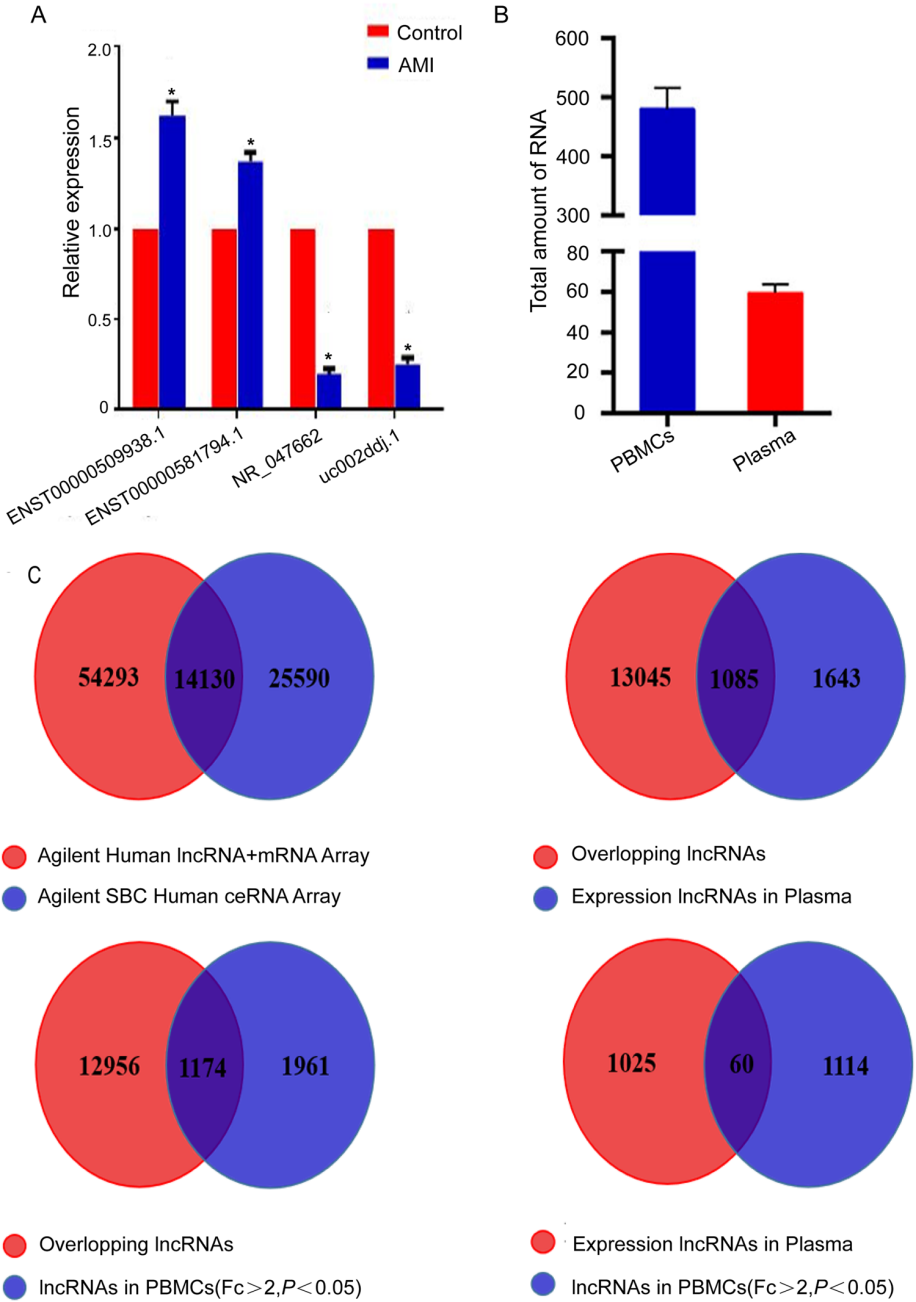
(**A**) qRT-PCR validation of 4 selected lncRNAs in study participants. Compared to matched controls, the expression of 4 lncRNAs, ENST00000509938.1, ENST00000581794.1, NR_047662.2, and uc002ddj.1, was significantly different between AMI and controls(*p < 0.05). (**B**) The amount of RNA in PBMCs and plasma was different (*p < 0.05). (**C**) Details of two chips and overlapping lncRNAs.

To validate the microarray data, we used qRT-PCR to detect the expression of lncRNAs (uc002ddj.1, ENST00000509938.1, ENST00000581794.1 and NR_047662.2) in PBMCs. We assessed the expression levels of the four selected lncRNAs from AMI patients (*n* = 50) and matched controls (*n* = 50) **(**Table 5**)**. Through our confirmation, ENST00000509938.1 was the most significantly increased (1.62-fold), followed by ENST00000581794.1 (1.37-fold). In addition, uc002ddj.1 (0.25-fold) and NR_047662.2 (0.2-fold) were significantly decreased (Fig. 6A). Convincingly, these results were consistent with the microarray chip analysis results.

**Table 5:** Clinical and phenotypical characteristics of 50 patients and 50 matched controls.

Characteristic	Matched controls	AMI patients
Age, years	52.8 ± 6.14	53.8 ± 5.22
BMI, kg/m^2^	23.7 ± 3.05	26.9 ± 3.44
Systolic blood pressure, mmHg	123.6 ± 14.31	118.1 ± 20.43
Diastolic blood pressure, mmHg	83 ± 9.75	79.5 ± 16.11
Heart rate, beats per minute	71 ± 7.14	80 ± 9.70
Creatinine, umol/L	80.4 ± 10.41	77.43 ± 17.10
Glucose, mmol/L	4.96 ± 0.27	7.44 ± 2.96*
TG, mmol/L	1.81 ± 0.5	2.61 ± 2.49*
TC, mmol/L	3.86 ± 0.87	4.21 ± 0.64*
LDL, mmol/L	3.19 ± 0.21	3.51 ± 0.54*
HDL, mmol/L	0.82 ± 0.11	1.21 ± 0.04*

The data are presented as the mean ± standard deviation. t-test, *p < 0.05 compared with matched controls.

### Differences in lncRNA expression between PBMCs and plasma

To express the level of nucleic acids more intuitively and conveniently in blood, we not only evaluated the microarray chips with PBMCs but also with plasma. Undoubtedly, the difference is obvious between PBMCs and plasma in terms of the quality of RNA. The abundance of RNA in PBMCs was higher than that in plasma (*p* < 0.05) (Fig. 6B). There were 14,130 overlapping lncRNAs between the two chips (Fig. 6C). Among the 14,130 total overlapping genes, 1174 lncRNAs were found to be differentially expressed in PBMCs with Fc>2 and *p* < 0.05, and 1085 lncRNAs were expressed in plasma. Among the two levels, we found 60 lncRNAs not only expressed in plasma but also significantly differentially expressed in PBMCs. The 60 lncRNAs in AMI patients are presented in Supplementary Table 5.

## Discussion

There is no doubt that lncRNAs have become important factors in biology; however, the majority of lncRNAs have not yet been researched. In this study, we described the lncRNA and mRNA expression profiles of clinical samples from 9 AMI patients and 9 controls via microarray analysis. To the best of our knowledge, this is the first study to identify differentially expressed lncRNAs in two sample types: PBMCs and plasma. Using a comprehensive analysis, we demonstrated that in AMI, not only mRNA expression but also lncRNA expression can be dysregulated. Functional coexpression analysis indicated that some of the differentially expressed lncRNAs and mRNAs might play crucial regulatory roles in several mechanisms previously implicated in the pathogenesis of AMI. Importantly, many of these altered lncRNAs are linked to bioprocesses associated with the inflammatory response, oxidative stress and apoptosis. Thus, we suggest that these altered lncRNAs might play indispensable roles as AMI biomarkers.

In PBMCs, a total of 2677 upregulated and 458 downregulated lncRNAs were identified to be significantly and differentially expressed; moreover, 1168 mRNAs were upregulated and 1334 mRNAs were downregulated between AMI patients and controls. In plasma, we found 41 upregulated and 51 downregulated lncRNAs that were differentially expressed, as well as 9 mRNAs that were upregulated and 9 mRNAs that were downregulated among the two groups. After analysing the data from the two chips, we obtained 14,130 overlapping lncRNAs. Among them, we found that 60 lncRNAs were differentially expressed in both PBMCs and plasma. The details of the plasma lncRNAs varied among coronary heart disease (CHD) patients, not only in our research but also in another report. Yang^14^ detected 174 lncRNAs that were upregulated, whereas 91 were downregulated according to Fc>2 and *p* < 0.01 using the Human LncRNA Array V2.0 (8660 K, Arraystar). Similarly, several studies have investigated the relationship between lncRNAs and AMI through expression profiling in myocardial infarction (MI) mice, but the findings of novel heart-specific lncRNAs were also diverse. For example, Ounzain^26^ found 143 upregulated and 291 downregulated lncRNAs; however, 301 upregulated and 341 downregulated lncRNAs were reported in another study^27^. One might wonder why this phenomenon exists, and there are some possible reasons that can explain it. In addition to the diversity of the clinical and demographic characteristics of the enrolled samples, the amount of RNA expression in plasma, PBMCs and tissue is tremendously diverse, even when the RNA quantity accords the standard of the microarray chip. Another reason is that the nucleic acid source in plasma, PBMCs and tissue differs. Unlike in plasma and tissue, nucleic acids released through ruptured cardiomyocytes are the main origin in plasma.

To date, most lncRNA functions have not been well annotated; therefore, GO and KEGG pathway and disease analyses have played indispensable roles in forecasting the functional relations among the coexpressed mRNAs and diseases. The GO analyses divided mRNAs into several functional modules, which are related to cardiac conduction, cellular response to calcium ion and cardiac muscle cell action potential involved in contraction, indicating the validity of the microarray. The KEGG analyses revealed that the mTOR signalling pathway, circadian rhythm, ARVC, the metabolism pathway, mitral valve diseases and QT interval were important pathways. All of these KEGG pathways participate in the process of AMI, providing evidence for the precision of the microarray. Then, we identified the top 20 upregulated and downregulated lncRNAs and their functional links. Corresponding to the dysregulation of lncRNAs in AMI, the most enriched pathways related to AMI were the PPAR signalling pathway, NF-kB signalling pathway, p53 signalling pathway and JAK/STAT signalling pathway. According to previous studies, it is important in cytokine regulation for PPAR^25,28–31^ to inhibit atherosclerosis and regulate glucose metabolism and lipid metabolism. Zhou *et al*.^31^ reported that myocardial apoptosis is associated with PPAR-γ following AMI in a rat model. The NF-kappa B signalling pathway^32,33^, p53 signalling pathway^32,34^ and JAK2/STAT3 signalling pathway^29,35–37^ play essential roles in CHD and also have significant effects on the inflammatory response, oxidative stress and apoptosis. During ischaemic injury, Leung *et al*.^38^ reported that the presence of CD4-expressing T cells, TNF-α and IL-10 were all in response to neoantigen inflammation. Regarding the role of the NF-kB signalling pathway, we hypothesized that its participation in the pathogenesis of AMI may also be related to CD4-expressing T cells, TNF-α and IL-10 in myocardial ischaemia and infarction. Next, we analysed the coexpression network between altered lncRNAs and mRNAs. We specifically chose the 4 lncRNAs, namely, uc002ddj.1, NR_047662.2, ENST00000509938.1, and ENST00000581794.1, based on their coexpressed mRNAs, which are related to the occurrence and development of AMI. For example, NEXN is known as an actin-binding protein that is associated with dilated cardiomyopathy^39^, hypertrophic cardiomyopathy^40^, CAD^41^ and atherosclerosis^42^. León *et al*.^43^ reported that KANSL1 microduplication, in combination with the 22q11.2 deletion, is associated with an increased risk of CHD. Several studies revealed that PKD1 is not only associated with vascular endothelial growth factor (VEGF)^44^ but also exhibited differential expression in the myocardial tissue of a rat model^45^. Moreover, TAGAP^46^, AQP4^47,48^, EPHX2^49^ and GNB5^50,51^ all provided new insights into the molecular mechanisms through the inflammatory response and apoptosis pathways underlying cardiovascular disease regulation. However, this hypothesis should be further investigated. The STRING database adopts public data and combined with our microarray data, it was used to construct a PPI network that facilitates the creation of several subnetworks through the degree method. A PPI network was built to identify key correlated functional mRNAs by utilizing the coexpression network and GO annotations. As a result, we found 7 key mRNAs with connectivity degrees greater than 20, including EGFR, TGFB1, RAC2, AKT2, HDAC2, CXCL12 and STAT1. Most mRNAs are closely associated with positive regulation of blood vessel endothelial cell migration, mononuclear cell proliferation and chemotaxis, apoptosis, response to oxidative stress, mTOR signalling pathway and JAK/STAT signalling pathway. Similarly, the results need further research for hypothesis formulation and validation.

Currently, AMI exerts substantial burdens on society, and the diagnostic and prognostic cornerstones of the clinical assessment of AMI often have uncertainty^52,53^. It is urgent to develop more easily accessible and highly accurate blood-based biomarkers for the early detection of AMI. PBMCs mainly consist of monocytes and lymphocytes, which are important groups of cells in the innate immune system. A hallmark of the onset and development of CAD is the continuous and increasing monocyte adhesion to the endothelium. Previous studies have shown a pivotal role for PBMCs in the pathogenesis of CAD and atherosclerotic plaque progression^54^. In our study, it is also worth mentioning that the expression levels of the lncRNAs uc002ddj.1 and NR_047662 were decreased and those of ENST00000509938.1 and ENST00000581794.1 were increased in the setting of AMI, which identified previously non-reported novel lncRNAs with altered expression. Through further analysis, we found that the changes in the Fc direction of lncRNA expression were maintained but less than those in the microarray data. We considered that the reasons for the difference were as follows. First, clinical and individual differences must be considered. Second, the methods were slightly different. The microarray was performed according to the Agilent Gene Expression Analysis protocol, and qPCR validation was performed using the SYBR Green PCR Master Mix method. Last, the trend of changes was consistent, which was in accordance with the findings of other reports^23^. Increasingly, studies have proven that the stability of plasma miRNAs is primarily attributed to their packaging in microvesicles or the formation of complexes with miRNA-binding proteins^55,56^. It is not well known whether lncRNAs have similar properties to these described miRNAs. Nevertheless, increasing emphasis has been placed on lncRNAs that can serve as biomarkers in plasma, e.g., lncRNA H19^57^ for gastric cancer, lncRNA HULC^58^ for HCC, lncRNA CoroMarker for AMI^14^ and lncRNA LIPCAR^8^ for heart failure after MI. The differential lncRNAs identified in plasma still need to be further validated by large samples.

We must address some limitations of our study. First, data reliability is limited due to the small sample size for the microarrays. Additionally, we focused on predicting differentially expressed lncRNA and mRNA functions, not determining the molecular mechanism of these lncRNAs. Further experiments are needed. Finally, all participants in this study were from one hospital, and divergences between different regions and nationalities are inevitable. Therefore, validation should be carried out in more prospective and larger clinical trials.

## Conclusion

Our study analysed and elaborated the expression profiles of lncRNAs and mRNAs that previously have unreported bioinformation in PBMCs and plasma between AMI and matched controls using microarrays. The dysregulated lncRNAs and mRNAs were defined and screened out with the development of high-throughput microarrays, and the application of bioinformatics provided new insights into researching the underlying molecular mechanisms in AMI. In the future, on one aspect, it is necessary to validate the expression of the identified key lncRNAs and mRNAs in a multi-centre and larger-scale study. On the other hand, to understand the pathological mechanism of AMI, we need to further investigate the biological significance of AMI in animal or cell line models. As such, special lncRNAs and mRNAs can serve as diagnostic, prognostic and therapeutic biomarkers in AMI.

## Supplementary information

Supplementary Tables.

## Data Availability

All data relevant to the study are included in the article or uploaded as supplementary information. No additional data are available.
