# Supplementary material for: Construction and analysis for differentially expressed long non-coding RNAs and mRNAs in acute myocardial infarction
Source: Sci Rep. 2020 Apr 24;10:6989. doi: 10.1038/s41598-020-63840-9 (PMC7181872; doi:10.1038/s41598-020-63840-9)
Supplement: Supplementary file 1 — Supplementary Tables. [file 41598_2020_63840_MOESM1_ESM.docx]

**Construction and analysis for differentially expressed long non-coding RNAs and mRNAs in acute** **myocardial infarction**

Ning Song^1*^, Xiang-Mei Li^1*^, Jun-Yi Luo^1,2^, Hui Zhai^1,2^, Qian Zhao^1,2^, Xin-Rong Zhou^1^, Fen Liu^1,2^, Xue-He Zhang^1^, Xiao-Ming Gao^1,2,3#^, Xiao-Mei Li^1,2#^ and Yi-Ning Yang^1,2#^

^1^ State Key Laboratory of Pathogenesis, Prevention and Treatment of High Incidence Diseases in Central Asia, Department of Cardiology, First Affiliated Hospital of Xinjiang Medical University, Urumqi, China.

^2^ Xinjiang Key Laboratory of Cardiovascular Disease Research, Clinical Medicine Institute, The First Affiliated Hospital of Xinjiang Medical University, Urumqi, China.

^3^ Xinjiang Key Laboratory of Medical Animal Model Research, Clinical Medical Research Institute of Xinjiang Medical University, Urumqi, China.

*These authors contributed equally to this work.

#Corresponding author. Email: [yangyn5126@163.com](mailto:yangyn5126@163.com), [lixm505@163.com](mailto:lixm505@163.com), xiaomingao2017@163.com.

**Corresponding authors:**

Prof. Yi-Ning Yang

Department of Cardiology, First Affiliated Hospital of Xinjiang Medical University, 137 Liyushan South Road, Urumqi, 830054, China.

Email: [yangyn5126@163.com](mailto:yangyn5126@163.com). Tel: 86-991-4361690. Fax: 86-991-4365330.

Prof. Xiao-Mei Li

Department of Cardiology, First Affiliated Hospital of Xinjiang Medical University, 137 Liyushan South Road, Urumqi, 830054, China.

E-mail: [lixm505@163.com](mailto:lixm505@163.com). Tel: 86-991-4361690. Fax: 0991-4362611.

Prof. Xiao-Ming Gao

State Key Laboratory of Pathogenesis, Prevention and Treatment of High Incidence Diseases in Central Asian, 137 Liyushan South Road, Urumqi, 830054, China.

E-mail: [xiaomingao2017@163.com](mailto:xiaomingao2017@163.com). Tel: 86-991-4362844. Fax: 86-991-4362844.

**Supplementary Table 1. The 20 of distinctively expressed lncRNAs in the** **PBMCs of AMI**

| **lncRNA ID** | ***p*** | **FC** | **Regulation** | **Probe ID** | **Chr** | **Start** | **End** | **class** |
| --- | --- | --- | --- | --- | --- | --- | --- | --- |
| ENST00000603472.1 | 0.003658 | 22.39658 | down | p37607_v4 | 4 | 1.52E+08 | 1.52E+08 |  |
| TCONS_00006729 | 0.025838 | 8.095924 | up | p21975 | 3 | 1.88E+08 | 1.88E+08 | Intergenic |
| ENST00000587325.1 | 0.031565 | 7.680118 | up | p6995 | 17 | 68014251 | 68069423 | Intergenic |
| ENST00000573315.1 | 0.006265 | 7.66713 | up | p6253 | 16 | 3051300 | 3052017 | Intergenic |
| TCONS_00012852 | 0.06847 | 7.519615 | up | p23866 | 7 | 1.42E+08 | 1.42E+08 | Intergenic |
| uc002ywy.3 | 0.087012 | 7.062308 | up | p26170 | 21 | 39645397 | 39647443 | Intronic |
| TCONS_00022804 | 0.016024 | 7.028577 | up | p19348 | 14 | 77541495 | 77542669 | Intergenic |
| TCONS_00004284 | 0.012967 | 6.939736 | up | p20855 | 2 | 64503034 | 64503985 | Intergenic |
| TCONS_00004804 | 0.011987 | 6.788351 | up | p21130 | 2 | 64501030 | 64550940 | Intergenic |
| ENST00000563515.1 | 0.027712 | 6.187538 | up | p6219 | 16 | 90182607 | 90192430 | Intergenic |
| XR_158932.2 | 0.012315 | 6.028117 | down | p30363 | 19 | 35167186 | 35168486 | Divergent |
| ENST00000474711.1 | 0.008801 | 5.953841 | up | p11463 | 3 | 1.1E+08 | 1.1E+08 | Intergenic |
| NR_103548.1 | 0.055221 | 5.89427 | up | p40728_v4 | 5 | 90598802 | 90610219 | - |
| ENST00000607744.1 | 0.016875 | 5.811808 | up | p37392_v4 | 3 | 37223597 | 37224225 | - |
| ENST00000537498.1 | 0.006375 | 5.714914 | up | p6208 | 16 | 89112579 | 89119373 | Intergenic |
| ENST00000534505.1 | 0.004636 | 5.714103 | up | p2799 | 11 | 65513199 | 65539056 | Intergenic |
| TCONS_00022562 | 0.082375 | 5.691758 | up | p19483 | 14 | 77426017 | 77432564 | Intergenic |
| XR_244991.2 | 0.037466 | 5.614318 | up | p39695_v4 | 2 | 40738648 | 40772522 | - |
| RNA33661\|snoRNA_scaRNA_257_80 | 0.021204 | 5.569618 | down | RNA33661\|snoRNA_scaRNA_257_80 |  |  |  | - |
| TCONS_00018817 | 0.019677 | 5.542457 | down | p18209 | 10 | 1.13E+08 | 1.13E+08 | Intergenic |

**Supplementary Table 2. The 20 of distinctively expressed mRNAs in the PBMCs of AMI**

| **Probe Name** | ***p*** | **FC** | **Regulation** | **GeneSymbol** | **Ensembl ID** |
| --- | --- | --- | --- | --- | --- |
| A_23_P97141 | 0.010527 | 9.488385 | down | RGS1 | ENST00000498352 |
| A_23_P258912 | 0.047253 | 9.237818 | up | MYOM2 | ENST00000520298 |
| A_23_P321307 | 0.061656 | 9.019884 | up | ADAMTS2 | ENST00000274609 |
| A_23_P216225 | 0.034386 | 7.149013 | down | EGR3 | ENST00000317216 |
| A_24_P14367 | 0.051885 | 6.404797 | down | PTBP1 | ENST00000394601 |
| A_24_P303091 | 0.099202 | 6.317447 | down | CXCL10 | ENST00000306602 |
| A_32_P225816 | 0.005172 | 6.132976 | up | PRDM16 | ENST00000270722 |
| A_23_P99360 | 0.007351 | 5.874894 | up | TRIM13 | ENST00000378182 |
| A_24_P35400 | 0.01326 | 5.773292 | down | SARDH | ENST00000422262 |
| A_33_P3409765 | 0.021149 | 5.61409 | up | MGAM |  |
| A_21_P0008272 | 0.007045 | 5.611445 | up | LOC100506999 | ENST00000553785 |
| A_33_P3865368 | 0.017369 | 5.533775 | up | LOC254896 |  |
| A_21_P0011832 | 0.027523 | 5.394102 | up | lnc-IL1R2-2 |  |
| A_32_P456318 | 0.013631 | 5.27644 | up | SRSF12 | ENST00000452027 |
| A_23_P329261 | 0.026125 | 5.266453 | up | KCNJ2 | ENST00000243457 |
| A_23_P15357 | 0.041526 | 5.226111 | down | LGALS3BP | ENST00000587251 |
| A_23_P114008 | 0.005139 | 5.205253 | up | TM4SF20 | ENST00000304568 |
| A_33_P3351566 | 0.004605 | 5.125072 | down | ETNK1 | ENST00000335148 |
| A_33_P3343175 | 0.129499 | 5.067863 | down | CXCL10 | ENST00000306602 |
| A_23_P48414 | 0.024865 | 5.066662 | down | CCNA1 | ENST00000255465 |

**Supplementary Table 3. The 20 of distinctively expressed lncRNAs in the plasma of AMI**

| **lncRNA ID** | ***p*** | **FC** | **Regulation** | **Probe ID** | **Chr** | **Start** | **End** | **class** |
| --- | --- | --- | --- | --- | --- | --- | --- | --- |
| NONHSAT135822 | 0.027293757 | 5.496381089 | down | LNCV6_125794 | M | 154486041 | 154486584 | exonic_sense |
| lnc-RP11-497E19.2.1-11:1 | 0.023053681 | 3.893625179 | down | LNCV6_61195 | 14 | 2885545 | 2887007 | intronic_antisense |
| lnc-GCNT1-4:1 | 0.00731686 | 3.461577451 | down | LNCV6_80896 | 9 | 67094931 | 67095930 | intergenic |
| NONHSAT128977 | 0.042489109 | 3.414642097 | down | LNCV6_125552 | 8 | 16057472 | 16057622 | intergenic |
| lnc-SLC15A4-24:1 | 0.005852646 | 3.265836196 | down | LNCV6_109699 | 12 | 217250 | 217401 | intergenic |
| lnc-REG3G-6:1 | 5.55E-05 | 3.251088499 | down | LNCV6_69369 | 2 | 34920858 | 34982452 | exonic_sense |
| lnc-AC008073.6.1-1:1 | 0.020367792 | 2.716612354 | dP | LNCV6_37760 | 2 | 43016047 | 43018811 | intergenic |
| NONHSAT081867 | 0.025494267 | 2.333278273 | down | LNCV6_122252 | 21 | 648 | 1601 | intergenic |
| ENST00000585691 | 0.033617453 | 2.267248122 | down | LNCV6_31719 | 18 | 57531769 | 57532355 | intronic_sense |
| ENST00000515896 | 0.021034991 | 2.232016599 | down | LNCV6_35352 | Y | 58850530 | 58850641 | intronic_antisense |
| NR_120654 | 0.003590195 | 2.230486308 | down | LNCV6_56530 | 10 | 38775835 | 38782990 | exonic_sense |
| NONHSAT135828 | 0.014567683 | 2.210269766 | down | LNCV6_125920 | M | 10197228 | 10198481 | intergenic |
| NR_104139 | 0.019342657 | 2.210269766 | up | LNCV6_29705 | 16 | 39833285 | 39833793 | exonic_antisense |
| ENST00000552835 | 0.013750975 | 2.136145427 | down | LNCV6_109648 | 12 | 80147076 | 80147521 | exonic_antisense |
| lnc-ABCA10-2:1 | 0.000322212 | 2.136145427 | up | LNCV6_63393 | 17 | 184378207 | 184379842 | intronic_antisense |
| lnc-SYNPR-2:9 | 0.024718621 | 2.111142467 | down | LNCV6_73132 | 3 | 96617184 | 96618062 | exonic_sense |
| ENST00000612732 | 0.046862051 | 2.041872241 | down | LNCV6_35347 | 22 | 13838177 | 13843136 | intergenic |
| lnc-SLC10A3-1:1 | 0.049126881 | 2.022711043 | down | LNCV6_94881 | X | 18788664 | 18794289 | intergenic |
| lnc-WRNIP1-24:4 | 0.019661057 | 2.022711043 | up | LNCV6_76638 | 6 | 52904976 | 52905077 | exonic_sense |
| lnc-RHOD-2:1 | 0.041107508 | 1.980938589 | down | LNCV6_57764 | 11 | 40322990 | 40331168 | exonic_sense |

**Supplementary Table 4. The 18 of distinctively expressed mRNAs in the plasma of AMI**

| **Probe Name** | ***p*** | | **FC** | **Regulation** | **GeneSymbol** | **Ensembl ID** |
| --- | --- | --- | --- | --- | --- | --- |
| LNCV6_139424 | 0.023560072 | 1.702146142 | | up | PPM1M | 132160 |
| LNCV6_139552 | 0.001619636 | 1.904689211 | | up | PA2G4 | 5036 |
| LNCV6_144647 | 0.020192528 | 2.147766351 | | down | OR7A10 | 390892 |
| LNCV6_137980 | 0.035274525 | 1.685690116 | | up | KRTAP10-3 | NA |
| LNCV6_128856 | 0.023761057 | 4.570774768 | | down | MTRNR2L6 | 100463482 |
| LNCV6_132622 | 0.02607644 | 1.523576828 | | down | ZKSCAN2 | 342357 |
| LNCV6_139193 | 0.020057502 | 1.566116577 | | down | FSTL1 | 11167 |
| LNCV6_137595 | 0.016521455 | 1.774445741 | | down | COL27A1 | 85301 |
| LNCV6_128888 | 0.004568753 | 1.755852372 | | up | ANKS4B | 257629 |
| LNCV6_89551 | 0.028709093 | 4.122871091 | | down | MTRNR2L8 | NA |
| LNCV6_61444 | 0.002640268 | 2.298139181 | | up | PLD4 | 122618 |
| LNCV6_131123 | 0.023631605 | 1.870338753 | | up | OR2V2 | 285659 |
| LNCV6_97219 | 0.015311542 | 4.921656845 | | down | MTRNR2L2 | 100462981 |
| LNCV6_141164 | 0.001131094 | 1.665596865 | | down | ZBTB45 | 84878 |
| LNCV6_144540 | 0.012837565 | 1.544993765 | | up | STIP1 | 10963 |
| LNCV6_121916 | 0.004656482 | 1.514296775 | | down | EEF1B2 | 1933 |
| LNCV6_139144 | 0.029524638 | 1.525092196 | | up | THOC7 | 80145 |
| LNCV6_140053 | 0.016717858 | 1.766944045 | | down | CALM3 | 808 |

**Supplementary Table 5. The 60 of overlopping lncRNAs in AMI patients**

| **lncRNA ID** | ***p*** | **FC** | **Regulation** | **Probe ID** | **Chr** | **Start** | **End** | **class** |
| --- | --- | --- | --- | --- | --- | --- | --- | --- |
| ENST00000563613.1 | 0.011426 | 3.8963 | up | p5921 | 16 | 19354968 | 19413015 | Intergenic |
| ENST00000569459.1 | 0.050254 | 3.116457 | up | p5971 | 16 | 30488500 | 30500674 | Antisense |
| ENST00000451730.1 | 0.006881 | 3.098138 | up | p9414 | 2 | 1.72E+08 | 1.72E+08 | Antisense |
| ENST00000566457.1 | 0.052253 | 2.97619 | down | p15618 | 8 | 22536525 | 22541522 | Intergenic |
| ENST00000587412.1 | 0.046578 | 2.91856 | up | p8707 | 19 | 36451413 | 36454965 | Intergenic |
| ENST00000442967.1 | 0.044725 | 2.882319 | up | p10201 | 2 | 2E+08 | 2E+08 | Intronic |
| ENST00000449730.1 | 0.046677 | 2.852089 | up | p16609 | 9 | 1.21E+08 | 1.21E+08 | Intergenic |
| ENST00000425094.1 | 0.088679 | 2.843841 | up | p4124 | 13 | 1.12E+08 | 1.12E+08 | Antisense |
| ENST00000427229.1 | 0.030612 | 2.823901 | up | p1936 | 10 | 45642841 | 45645173 | Intergenic |
| ENST00000563267.1 | 0.030123 | 2.798724 | up | p6136 | 16 | 80560735 | 80574850 | Divergent |
| ENST00000448734.1 | 0.04319 | 2.774992 | down | p9228 | 2 | 95690937 | 95692454 | Antisense |
| ENST00000599803.1 | 0.008685 | 2.703683 | up | p8443 | 19 | 53771122 | 53774185 | Divergent |
| ENST00000447876.1 | 0.032731 | 2.656924 | up | p9259 | 2 | 1.05E+08 | 1.05E+08 | Intergenic |
| ENST00000446989.1 | 0.077827 | 2.647772 | up | p4136 | 13 | 1.15E+08 | 1.15E+08 | Intergenic |
| ENST00000497573.1 | 0.007281 | 2.635113 | up | p11825 | 3 | 63091650 | 63099571 | Intergenic |
| ENST00000543182.1 | 0.071428 | 2.592679 | up | p34195_v4 | 11 | 1.15E+08 | 1.15E+08 | Intronic |
| ENST00000555107.1 | 0.008147 | 2.592178 | up | p4440 | 14 | 37116287 | 37128006 | Antisense |
| ENST00000520418.1 | 0.0423 | 2.570871 | up | p16145 | 8 | 41518825 | 41521766 | Antisense |
| ENST00000586784.1 | 0.013245 | 2.52597 | up | p8639 | 19 | 28284390 | 28289190 | Intergenic |
| ENST00000607109.1 | 0.022113 | 2.516733 | up | p38892_v4 | 19 | 43864836 | 43873122 |  |
| ENST00000571328.1 | 0.023776 | 2.509632 | up | p38896_v4 | 19 | 52197462 | 52205023 |  |
| ENST00000530792.1 | 0.047336 | 2.485877 | up | p2410 | 11 | 74919098 | 74952218 | Antisense |
| ENST00000416861.1 | 0.06804 | 2.481554 | up | p10267 | 2 | 2.21E+08 | 2.21E+08 | Intergenic |
| ENST00000563807.1 | 0.014457 | 2.47321 | up | p14184 | 6 | 45541082 | 45544507 | Intergenic |
| NR_046869.1 | 0.049323 | 2.472069 | up | p30068 | 13 | 79361453 | 79398991 | Intergenic |
| ENST00000505347.1 | 0.011614 | 2.471225 | up | p12256 | 4 | 19173957 | 19458617 | Intergenic |
| ENST00000577297.1 | 0.080638 | 2.438312 | up | p2970 | 11 | 1.19E+08 | 1.19E+08 | Divergent |
| ENST00000499096.2 | 0.011639 | 2.421268 | up | p13554 | 5 | 1.81E+08 | 1.81E+08 | Antisense |
| ENST00000515513.1 | 0.040628 | 2.394295 | up | p14028 | 5 | 1.74E+08 | 1.74E+08 | Intergenic |
| ENST00000606110.1 | 0.004271 | 2.391469 | up | p38483_v4 | 12 | 1.34E+08 | 1.34E+08 |  |
| ENST00000464125.1 | 0.082013 | 2.376767 | up | p11819 | 3 | 58592806 | 58620167 | Antisense |
| ENST00000414686.1 | 0.024348 | 2.368829 | down | p1078 | 1 | 1.01E+08 | 1.01E+08 | Intronic |
| ENST00000504506.1 | 0.145573 | 2.336247 | up | p13727 | 5 | 76384099 | 76427032 | Intergenic |
| ENST00000569137.1 | 0.011889 | 2.320384 | up | p5252 | 15 | 74163291 | 74165707 | Divergent |
| ENST00000578583.1 | 0.009932 | 2.270538 | up | p7929 | 18 | 19253588 | 19262522 | Intronic |
| ENST00000546264.1 | 0.110291 | 2.269321 | up | p3935 | 12 | 1.31E+08 | 1.31E+08 | Intronic |
| ENST00000437983.2 | 0.059127 | 2.236974 | up | p4016 | 13 | 37423502 | 37424183 | Intronic |
| NR_104672.1 | 0.019486 | 2.210751 | up | p40765_v4 | 5 | 1.12E+08 | 1.12E+08 |  |
| ENST00000556850.1 | 0.030232 | 2.207189 | up | p3706 | 12 | 57390042 | 57392225 | Antisense |
| ENST00000607415.1 | 0.015295 | 2.188513 | up | p37114_v4 | 2 | 3500726 | 3501199 |  |
| ENST00000423712.1 | 0.007281 | 2.180577 | up | p11015 | 22 | 35099116 | 35100876 | Intergenic |
| ENST00000443579.1 | 0.006501 | 2.173708 | up | p862 | 1 | 27852315 | 27857072 | Intergenic |
| ENST00000430978.1 | 0.09737 | 2.160529 | up | p4133 | 13 | 1.15E+08 | 1.15E+08 | Intergenic |
| ENST00000599129.1 | 0.101007 | 2.135669 | up | p8631 | 19 | 22605314 | 22608847 | Divergent |
| ENST00000454737.1 | 0.118842 | 2.116052 | up | p34924_v4 | 21 | 19181261 | 19183162 | Intronic |
| ENST00000567545.1 | 0.024652 | 2.104389 | down | p6363 | 16 | 31413885 | 31416020 | Antisense |
| ENST00000511677.1 | 0.059315 | 2.071799 | up | p2808 | 11 | 67653966 | 67658816 | Intergenic |
| ENST00000421642.1 | 0.040628 | 2.069643 | up | p10611 | 20 | 51288455 | 51307147 | Intergenic |
| ENST00000583794.1 | 0.084228 | 2.056209 | up | p34596_v4 | 18 | 22305907 | 22324842 | Intergenic |
| ENST00000435649.1 | 0.021555 | 2.054615 | up | p276 | 1 | 91297081 | 91317175 | Intergenic |
| ENST00000415556.1 | 0.138133 | 2.041029 | up | p7562 | 17 | 79899744 | 79905477 | Antisense |
| ENST00000557723.1 | 0.028677 | 2.040374 | up | p4858 | 14 | 66578348 | 66592565 | Intergenic |
| ENST00000599889.1 | 0.011268 | 2.038344 | up | p8888 | 19 | 58873950 | 58877958 | Antisense |
| ENST00000452532.1 | 0.101395 | 2.034823 | up | p17189 | X | 55934555 | 56041606 | Intergenic |
| ENST00000519793.1 | 0.066502 | 2.023839 | up | p15862 | 8 | 1.02E+08 | 1.02E+08 | Intergenic |
| ENST00000546738.1 | 0.046917 | 2.020282 | up | p3656 | 12 | 48405086 | 48419252 | Intergenic |
| ENST00000596286.1 | 0.008529 | 2.019916 | up | p8817 | 19 | 51517629 | 51517990 | Antisense |
| ENST00000521101.1 | 0.03901 | 2.012503 | up | p16101 | 8 | 29384828 | 29387840 | Intergenic |
| ENST00000580528.1 | 0.102321 | 2.003092 | down | p14935 | 7 | 45025060 | 45026560 | Intergenic |
| ENST00000433510.1 | 0.913059 | 1.030974 | up | p6908 | 17 | 46713653 | 46724385 | Intergenic |
